# LGR5 expression is controled by IKKα in basal cell carcinoma through activating STAT3 signaling pathway

**DOI:** 10.18632/oncotarget.8465

**Published:** 2016-03-30

**Authors:** Jiantao Jia, Ying Shi, Bin Yan, Deshen Xiao, Weiwei Lai, Yu Pan, Yiqun Jiang, Ling Chen, Chao Mao, Jian Zhou, Sichuan Xi, Ya Cao, Shuang Liu, Yongguang Tao

**Affiliations:** ^1^ Center for Medicine Research, Xiangya Hospital, Central South University, Changsha, Hunan, China; ^2^ Cancer Research Institute, Central South University, Changsha, Hunan, China; ^3^ Pathophysiology Department of Changzhi Medical College, Changzhi, Shanxi, China; ^4^ Key Laboratory of Carcinogenesis and Cancer Invasion, Ministry of Education, Hunan, China; ^5^ Key Laboratory of Carcinogenesis, Ministry of Health, Hunan, China; ^6^ Department of Pathology, Xiangya Hospital, Central South University, Changsha, Hunan, China; ^7^ Liver Surgery Department, Liver Cancer Institute, Zhongshan Hospital, Key Laboratory of Carcinogenesis and Cancer Invasion (Fudan University), Ministry of Education, Fudan University, Shanghai, China; ^8^ Institute of Biomedical Sciences, Fudan University, Shanghai, China; ^9^ Thoracic Surgery Section, Thoracic and GI Oncology Branch, Center for Cancer Research, National Cancer Institute, Bethesda, MD, USA

**Keywords:** LGR5, IKKα, STAT3, BCC, EGF

## Abstract

Basal cell carcinomas (BCC) of the skin are the most common of human cancers. The noncanonical NF-κB pathway is dependent on IKKα. However, the role of IKKα in BCC has not been elucidated. We show here that IKKα is expressed in the nucleus in BCC and non-malignant diseases. Nuclear IKKα could directly bind to the promoters of inflammation factors and LGR5, a stem cell marker, in turn, upregulating LGR5 expression through activation of STAT3 signaling pathway during cancer progression. Activation of STAT3 signaling pathway contributes LGR5 expression in dependent of IKKα after the interplay between STAT3 and IKKα. Meanwhile knockdown of IKKα inhibits tumor growth and transition of epithelial stage to mescheme stage. Taken together, we demonstrate that IKKα functions as a bone fide chromatin regulator in BCC, whose promoted expression contributes to oncogenic transformation via promoting expression stemness- and inflammatory- related genes. Our finding reveals a novel viewpoint for how IKKα may involve in BCCs tumor progression in the inflammatory microenvironment.

## INTRODUCTION

G-Protein-coupled receptor GPR49, also known as LGR5, belongs to the leucine-rich repeat containing G-protein-coupled receptors (LGRs) structurally similar to glycoprotein hormone receptors including thyroid-stimulating hormone receptor, follicle-stimulating hormone receptor, and luteinizing hormone receptor, and functions as a marker of the crypt basal columnar stem cells along the gastrointestinal tract and of the bulge stem cells in the hair follicle [1, 2]. LGR5 is overexpressed in several types of cancer and can promote the growth/metastasis of colon tumor cells [3].

Nuclear factor (NF)-κB activation leads to a protumorigenic inflammatory microenvironment of various tumors [4]. The NF-κB pathway is tightly regulated by the IκB-kinase (IKK) complex, which consists of two catalytic subunits, IKKα and IKKβ, and a regulatory subunit, IKKγ [5]. Whereas, in most malignancies, the classical IKKβ/ IKKγ-dependent NF-κB activation controls key functions for tumor initiation, promotion and progression in tumors [6]. The noncanonical NF-κB pathway is dependent on IKKα, and the role of IKKα in noncanonical NF-κB pathway is more complex [7, 8]. Depending on the type of malignancy, IKKα can provide both tumor-promoting and tumor-suppressive mechanisms that are in most instances cell autonomous.

Signal transducer and activator of transcription 3 (STAT3) mediates a key intrinsic pathway promoting tumorigenesis [9]. While constitutive STAT3 activity had initially been attributed to deregulated growth factor signaling, recent studies have identified STAT3 as an important mediator of carcinogenesis driven by chronic inflammation, obesity and/or metabolism, cancer stem cells (CSCs) [9–15]. STAT3 is constitutively active and associated with poor clinical prognosis in several cancers and consequently, STAT3 is an attractive target for pharmacologic intervention in cancer patients [9, 16].

Basal cell carcinoma (BCC) and squamous cell carcinoma (SCC) and are two major types of skin cancer derived from keratinocytes [17]. Although the mortality attributable to BCC is not high, the disease is responsible for considerable morbidity, imposing a growing burden on healthcare services [18, 19]. In addition, IKKα reduction promotes chemical carcinogen- and ultraviolet B-induced skin carcinogenesis, and IKKα deletion in keratinocytes causes spontaneous skin SCCs, but not BCCs, in mice [20–24]. However, the role of IKKα in BCCs remains poorly known.

Here we show that IKKα is expressed in BCCs while down-regulates in SCCs of skin cancer. The physiological role of IKKα in BCCs is related with LGR5 expression. Our findings further confirm that IKKα directly binds to the LGR5 promoter in dependent of activating STAT3 signaling pathway. Our finding reveals a novel viewpoint for how IKKα may involve in BCCs tumor progression in the inflammatory microenvironment.

## RESULTS

### IKKα is dysregulated in skin-related tumors

**Figure 1:**
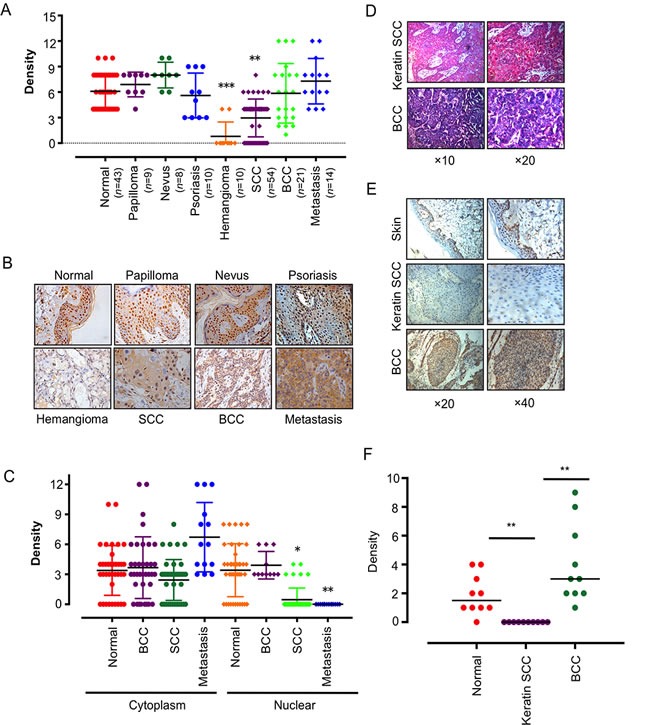
IKKα was dysregulated and delocalized in skin-related tumors **A.**. Anti- IKKα staining intensity was quantified in three microscopic fields for each tissues section and expression level of IKKα in skin-related diseases as indicated. *n*, number of analyzed samples. **B.**. A series of tissues samples was subjected to IHC with IKKα-specific antibody. The panels shown here were representative of IKKα staining in skin-related diseases as indicated. (Magnification: 40×.) **C.**. Distribution of IKKα in the cytoplasm and nucleus was quantified in BCC, SCC and metastasis. **D.** H & E staining was used to show different tissue type of BCC and keratin SCC. **E.** Immunohistochemical analysis was used to examine the level of IKKα in BCC and keratin SCCs. **F.** Expression level of IKKα in normal skin, keratin SCC and BCC as indicated. * *p* < 0.05, ** *p* < 0.01, *** *p* < 0.001.

We first analyzed IKKα expression and subcellular localization by immunohistochemistry (IHC) in a panel section of 10 hemangioma, 54 SCC, 21 BCC and 13 metastasis cancer in skin and 70 sections of normal noncancerous tissues. The normal tissues were subdivided into 43 normal skins from various tissues, 9 papilloma, 8 nevus and 10 psoriasis (Figure 1A). IKKα were significantly down-expressed in SCCs and silenced in hemangioma, while IKKα level did not change in normal skin tissues, noncancerous tissues in papilloma, nevus and psoriasis. Unexpectedly, we also did not find the changes of IKKα in BCC and metastasis tissues (Figure 1A and Figure 1B).

Interestingly, although strong nuclear localization of IKKα was detected in normal stratified epithelia and BCC, the IKKα staining appeared stranded in the cytoplasm in SCC and metastasis skin tissues (Figure 1B). The ratio of cytoplasm to nuclear increased from 0.89 in normal skin, to 1.82 in SCC and to 5.85 in metastasis (Figure 1C), indicating that down-regulation of IKKα in SCC and hemangioma and delocation of IKKα in SCC and metastasis may be associated with this critical step in tumor progression. It also hinted that nuclear IKKα might contribute to BCC carcinogenesis.

We noticed that about one fourth of SCCs did not detect IKKα expression, and we identified them as keratin SCC (Figure 1D). We further confirmed that keratin SCC was absence of IKKα as compared to BCC and normal skin (Figure 1E and 1F), indicating that the role of IKKα in skin cancer is dependent on the subtype of cancer.

### Inhibition of IKKα reduces LGR5 expression

**Figure 2:**
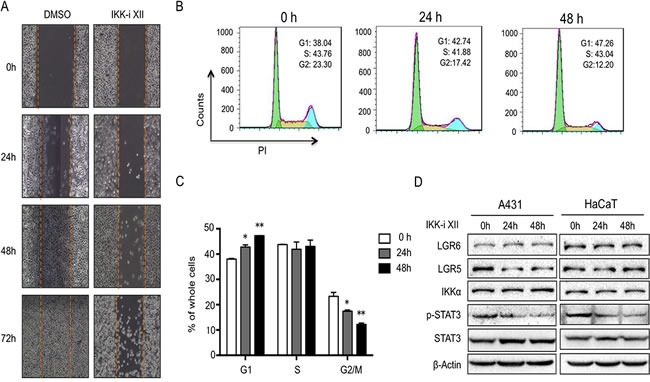
Inhibition of IKKα reduces LGR5 expression **A.**. A431cells with the treatment of IKKi-II were analyzed for their ability to migrate in a wound healing assay at indicated time points. **B.**. FACS analysis was used to detect cell cycle progression in A431 cells after the treatment of IKKi-II. **C.**. Statics of FACS analysis in A431 cells after the treatment of IKKi-II. * *p* < 0.05, ** *p* < 0.01. **D.**. A431 (Left) and HaCaT (Right) with treatment of IKKi-II were examined for the expression of LGR6, LGR5, IKKα, p-STAT3, STAT3 and β-actin by Western analysis.

To address potential link of IKKα with LGR5, we first treated A431 cells, derived from a human epidermal carcinoma of the vulva, with IKKα inhibitor. We found that IKK inhibitor XII (IKK-i XII), an ATP site-targeting inhibitor against IKK [25], inhibited cell migration (Figure 2A). Using FACS, we found that IKK-i XII promotes G1 stage and decreased G2/M stages in A431 cells, indicating that inhibition of IKK in A431 cells might block cell cycle progression (Figure 2B and 2C).

After we treated A431 cells with IKK-i XII, we found that LGR5 level and phospharylated STAT3 (Y705) decreased while other proteins including LGR6, STAT3 and IKKα remained the same level (Figure 2D *left*). Similar findings were found in HaCaT cells, an immortalized human keratinocytes, after the treatment of IKK-i XII (Figure 2D *right*), indicating that IKKα might involve in the regulation of LGR5.

### Knockdown of IKKα decreases LGR5 expression

**Figure 3:**
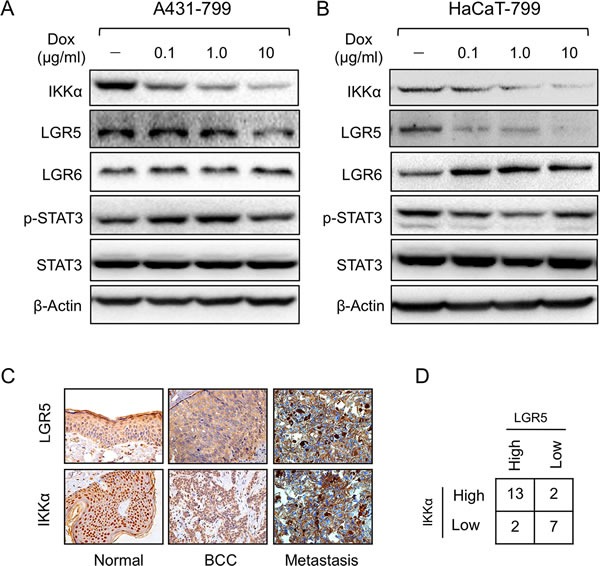
Knockdown of IKKα decreased LGR5 expression A431 **A.** and HaCaT **B.** cells were examined for the expression of proteins as indicated by Western analysis after an inducible knockdown Tet-on system of IKKα the cells were stably transfected. **C.**. Immunohistochemical analysis was used to examine the level of LGR5 protein in skin-related diseases. LGR5 was expressed at high levels whereas IKKα was also expressed at higher levels in skin BCC tissues (Tumor, Metastasis). (Magnification: 40×.) **D.**. Summary of LGR5 and IKKα protein levels in 35 tissue samples. Fisher's test, two-tailed *p* < 0.01.

To further identify link of IKKα with LGR5, we established an inducible knockdown of IKKα with RNAi that were selected from three shRNA sequences in both HaCaT and A431 cells. We first confirmed the inducible system knockdowned IKKα specially and efficiency (Supplementary Figure S1). After HaCaT cells were treated with different concentrations of doxycline (Dox) for 48 h, we found that the inducible knockdown system successfully reduced IKKα protein to less than 10% (Figure 3A). Meanwhile, LGR5 protein level also significantly decreased as company with knockdown of IKKα. Other proteins including p-STAT3, total STAT3 and LGR6 remained the same level (Figure 3A). Similar findings were found in A431 cells that were stable expression the inducible knockdown of IKKα system (Figure 3B), indicating that a strong correlation might exist in IKKα and LGR5.

To address the role of other members of IKK complex, we detected LGR5 expression after the depletion of IKKβ and IKKγ with shRNA respectively. LGR5 expression did not change significantly after the depletion of IKKβ using two separate shRNA sequences (Left panel of Supplementary Figure S2), meanwhile LGR5 expression slightly decreasead after complete depletion of IKKγ (Right panel of Supplementary Figure S2). Data indicated that both IKKβ and IKKγ was not involved in the regulation of LGR5 expression.

To further confirm the correlation between IKKα and LGR5, we analyzed LGR5 expression in skin BCC and metastasis biopsies using IHC. LGR5 protein expression was greatly increased in BCC tumor samples as well as metastasis (Figure 3C). The evaluation of IKKα and LGR5 protein levels of all 35 biopsies conformed the positive correlation between IKKα and LGR5 (*p* < 0.01) (Figure 3D). Taken together, these results suggest that IKKα might be a major activating signal for LGR5 expression in BCC.

### Inflammatory factors activate STAT3 signaling pathway that is controlled by IKKα

**Figure 4:**
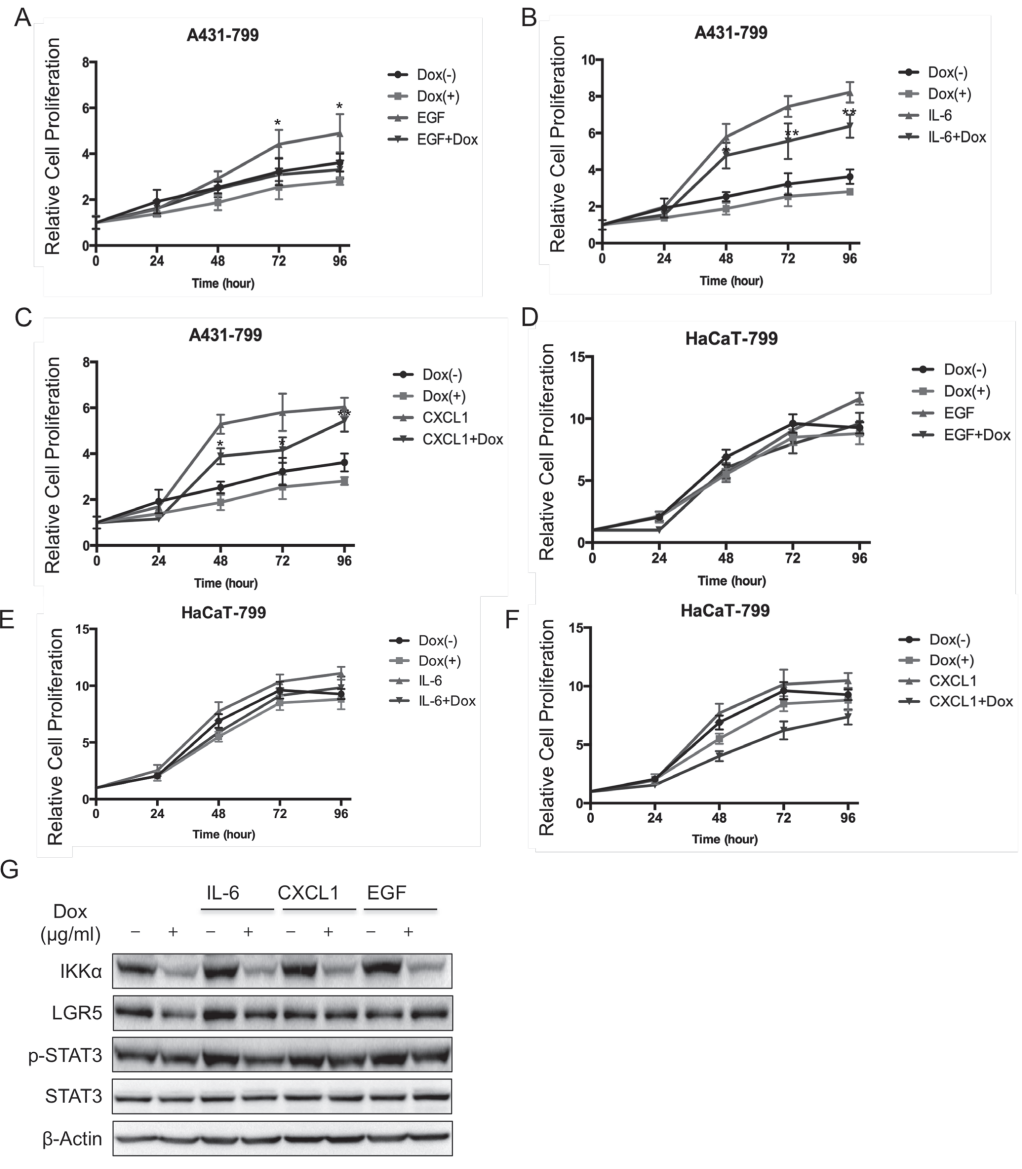
Inflammatory factors activated STAT3 signaling pathway that was controlled by IKKα The MTT assay was performed to assess cell viability in A431 cells that were stably transfected with an inducible Tet-on IKKα shRNAs and the cells were treated with EGF **A.**, IL-6 **C.**, and Cxcl1 **E.**. The MTT assay was performed to assess cell viability in HaCaT cells that were stably transfected with an inducible Tet-on IKKα shRNAs and the cells were treated with EGF **B.**, IL-6 **D.**, and Cxcl1 **F.**. **G.**. A431 cells with an inducible knockdown Tet-on system of IKKα were examined for the expression of proteins as indicated by Western analysis after the treatment of inflammatory factors for 24 h. * *p* < 0.05, ** *p* < 0.01.

Since the JAK-STAT pathway is possibly involved in BCC pathogenesis, EGF, IL-6 and Cxcl1 trigger STAT3 signaling pathway [26]. We first treated cells with inflammatory factors, and found that EGF, IL-6 and Cxcl1 increased cell proliferation. Also knockdown of IKKα reduced cell growth in the absence or presence of EGF, Il-6 and Cxcl1 in A431 cells (Figure 4A, Figure 4C and Figure 4E) and in HaCaT cells (Figure 4B, Figure 4D and Figure 4F). Taken together, data indicates that IKKα involves in STAT3 signaling pathway.

Next, we analyzed the potential role of STAT3 signaling pathway in LGR5 expression. We treated A431 cells that IKKα was depleted in an inducible manner with inflammatory factors. We found that IKKα was depleted after the treatment of Dox in the absence and presence of IL-6, Cxcl1 and EGF. Meanwhile, LGR5 protein level slightly decreased after knockdown of IKKα in the presence of IL-6, Cxcl1 and EGF, indicating that STAT3 signaling pathway might involve in the regulation of LGR5 (Figure 4G). However both total STAT3 and phospharylated STAT3 at tyrosine 705 (p-STAT3) remained the same level (Figure 4G), indicating that STAT3 might increase directly LGR5 expression.

### Inhibiting STAT3 signaling pathway decreases LGR5 expression

**Figure 5:**
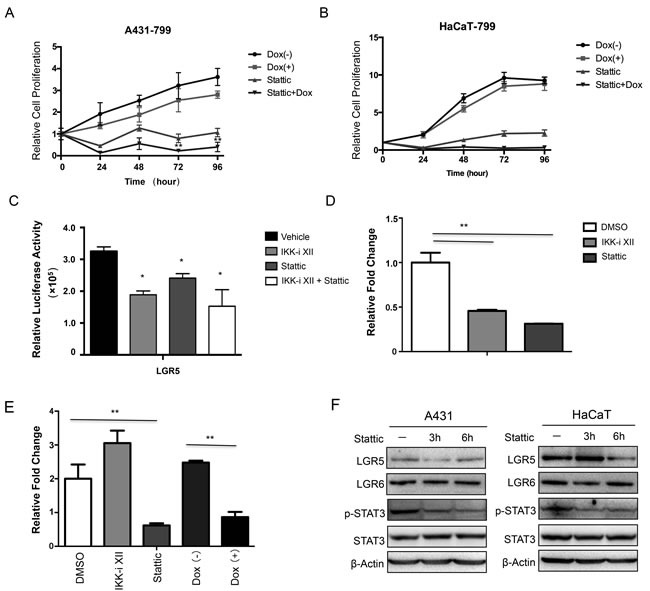
Activation of STAT3 signaling pathway was involved in the regulation of LGR5 expression The MTT assay was performed to assess cell viability in A431 cells **A.** and HaCaT cells **B.** that were stably that were stably transfected with an inducible Tet-on IKKα shRNAs and the cells were treated with Stattic. **C.**. A luciferase reporter assay was carried out to evaluate LGR5 promoter activity in 293 cells after the treatment of IKKi-II and/or Stattic. All promoter luciferase intensity was normalized to the pRL renilla luciferase control reporter. * *p* < 0.05, ** *p* < 0.01. **D.**. ChIP analysis in HaCaT cells and chemicals treatment indicated was performed to detect p-STAT3 binding to LGR5 gene. **E.**. ChIP analysis in A431 cells after Tet-on shIKKα transfected and chemicals treatment indicated was performed to detect p-STAT3 binding to LGR5 promoter gene. **F.**. A431 (Left) and HaCaT (Right) with treatment of Stattic were examined for the expression of selected proteins by Western analysis. ** *p* < 0.01

Stattic is a small molecule shown to selectively inhibit the activation of the STAT3 transcription factor by blocking phosphorylation and dimerization events. We found that Stattic decreased cell growth using an inducible knockdown of IKKα in both A431 and HaCaT cells, and we also showed that the combination of Stattic and knockdown of IKKα role in reducing cell growth in A431 cells (Figure 5A) and HaCaT cells (Figure 5B). Moreover, both IKKi-II and Stattic down-regulated significantly the LGR5 promoter transcription (Figure 5C).

Then we performed ChIP assay to address whether p-STAT3 involved in regulation of LGR promoter directly, we found that both IKKi-II and Stattic decreased the binding ability of p-STAT3 in the LGR5 promoter in HaCaT cells (Figure 5D and 5E). However, only IKKi-II could not decrease the binding ability of p-STAT3 in the LGR5 promoter in A431 cells (Figure 5E). Moreover, knockdown of IKKα decreased the binding of p-STAT3 to LGR5 promoter (Figure 5E). Lastly, we treated both HaCaT and A431 cells with Stattic as time indicated, both p-STAT3 phospharylation level and LGR5 protein level decreased (Figure 5F), indicating that activated STAT3 controlled LGR5 expression directly.

### IKKα directly targets to the inflammatory factors and LGR5

**Figure 6:**
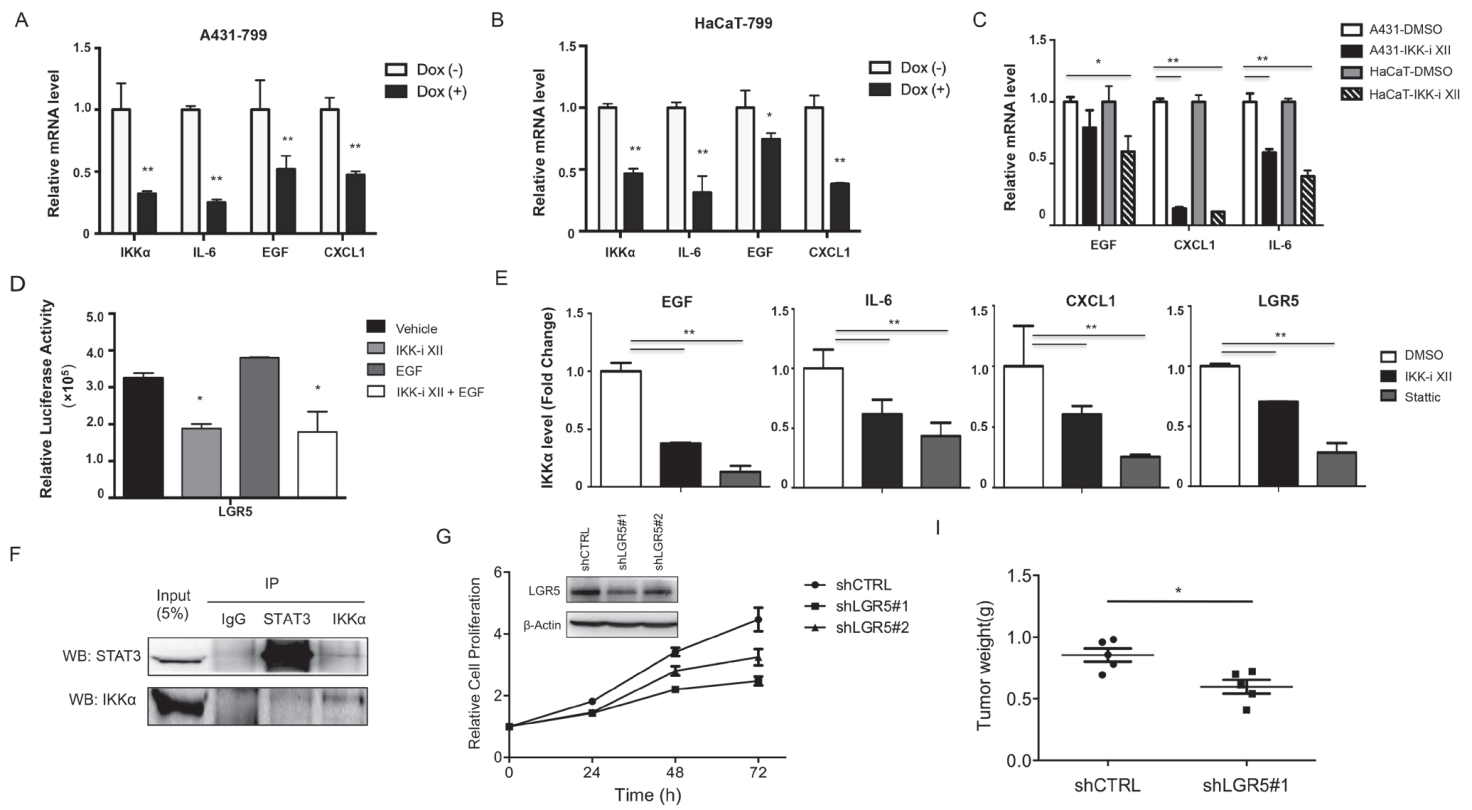
IKKα targeted to the inflammatory factors and LGR5 RT-PCR analysis was conducted to detect selected inflammatory factors using total RNA obtained from A431 cells **A.** and HaCaT cells **B.** after Tet-on shIKKα transfected. **C.** RT-PCR analysis was conducted to detect selected inflammatory factors using total RNA obtained from both HaCaT and A431 cells after the treatment of IKK-I II. **D.**. A luciferase reporter assay was carried out to evaluate LGR5 promoter activity in 293 cells. All promoter luciferase intensity was normalized to the pRL renilla luciferase control reporter. **E.**. ChIP analysis of A431 cells and chemicals treatment indicated was performed to detect IKKα binding to selected genes. **F.** Co-IP detected the intact complex of IKKα and STAT3 **G.** Cell proliferation in A431 was performed after the knockdown of LGR5. LGR5 protein levels, as detected by Western analysis, were shown in the inlet figure. **H.** A xenograft model of tumor weight was established in nude mice to evaluate the ability of knockdown of LGR5 in A431 cells and shCTRL cells to form cells. * *p* < 0.05, ** *p* < 0.01.

Since IKKα plays a critical role inflammation, we addressed whether IKKα could affect inflammatory factor directly. We detected mRNA level of EGF, IL-6 and Cxcl1 after the knockdown of IKKα in an inducible system. We found that IKKα mRNA reduced to less than 40% after the treatment of Dox for 48 h in A431 cells, and mRNA levels of EGF, IL-6 and Cxcl1 decreased significantly (Figure 6A). Similar findings were shown in HaCaT cells after knockdown of IKKα (Figure 6B). Furthermore, we treated both A431 and HaCaT cells with IKK-i II for 48 h, and we found that mRNA levels of EGF, Cxcl1 and IL-6 decreased significantly, while Cxcl1 mRNA level reduced to less than 10% (Figure 6C), indicating that IKKα involves in the control of these inflammatory factors.

To address whether IKKα links with LGR5 expression, we constructed a report gene of LGR5 that 1077 bp of LGR5 promoter was inserted into pGL4.16. After we transfected LGR5 reporter gene into 293 cells and treated the cells with the chemicals indicated for 72 h, and we found that both IKKi-II and Stattic decreased LGR5 promoter transcription while EGF slightly increased the transcription of LGR5 promoter (Figure 6D and Supplementary Figure S3). As IKKα could locates in nucleus and functions as a chromatin modifier [27, 28], we addressed whether IKKα involved in the regulation of inflammatory factors and LGR5 directly. We performed ChIP assay in A431 cells after the treatment of both IKKi-II and Stattic, we amplified the potential binding site of IKKα in the promoter of inflammatory genes and LGR5 as indicated in supplementary Figure S4 using real-time PCR. We found that IKKα bound to the promoter regions of EGF, IL-6 and CxCl1 and we also showed that IKKα was enriched in the promoter of LGR5 (Figure 6E), indicating that IKKα controls the inflammatory genes and LGR5 directly. On the basis of this, we wondered whether both IKKα and STAT3 formed an intact complex, we found that IKKα might interact with STAT3 in A431 cells (Figure 6F). To further address the role of LGR5 in skin carcinogenesis, we generated stable LGR5 knockdown in A431 cells using a set of shLGR5 lentivirus vectors (see ‘Material and Method’ section) (inlet of Figure 6G). The knockdown of LGR5 resulted in significantly reduced growth of A431 cells in culture (Figure 6G) and tumor weight after seeding 3 × 10^6^ of A431 cells (with or without LGR5 knockdown) into nude mice (Figure 6H).

### IKKα affects tumor growth *in vivo*

**Figure 7:**
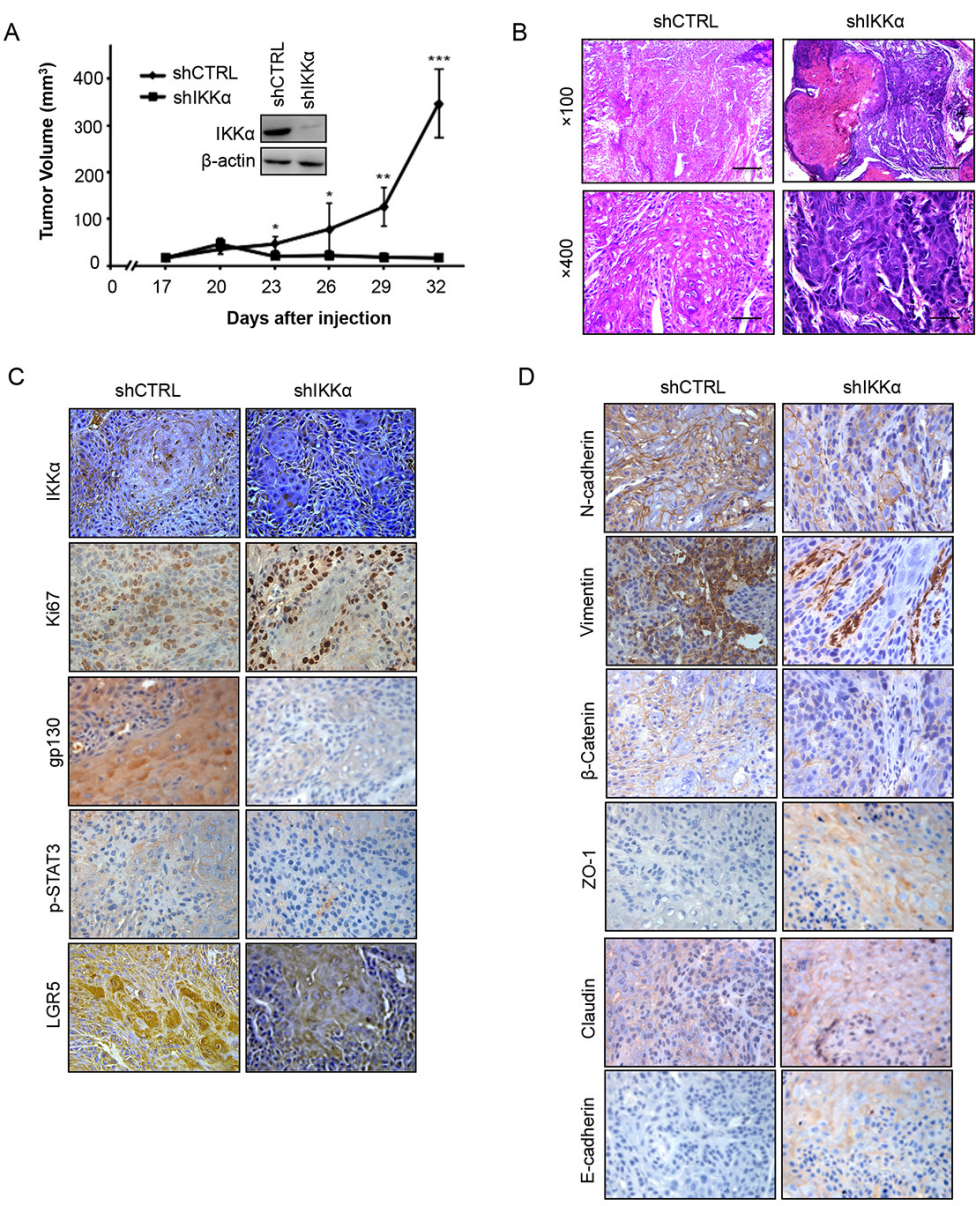
Inhibition of IKKα reduced tumor growth *in vivo* **A.**. A xenograft model of tumor growth was established in nude mice to evaluate the ability of knockdown of IKKα in A431 cells and shCTRL cells to form cells. IKKα protein levels, as detected by Western analysis, were shown in the inlet figure. * *p* < 0.05, * *p* < 0.01, *** *p* < 0.001. **B.**. Tumor status was analyzed by H & E staining. **C.**. Immunohistochemical analysis was used to examine the level of IKKα, Ki67, gp130, p-STAT3 and LGR5 in tumor samples from nude mice. (Magnification: 40×.) **D.**. Immunohistochemical analysis was used to examine the level of EMT markers as indicated in tumor samples from nude mice. (Magnification: 40×.).

To further validate the physiological role of IKKα in BCC carcinogenesis, we generated stable IKKα knockdown in A431 cells *in vivo*. We injected 3 × 10^6^ of A431 cells (with or without IKKα knockdown) into nude mice, and observed that IKKα depletion significantly impaired the tumor volume (Figure 7A), tumor formation (Figure S5A) and tumor weight (Figure S5B), while the body weight did not change significantly in either groups (Figure S5C). We confirmed the tumor formation using H & E staining (Figure 7B).

Next, using immunohistochemistry staining, we found that the levels of IKKα, Ki67, gp130, p-STAT3 and LGR5 decreased in biopsies from transplanted nude mice after the depletion of IKKα (Figure 7C). Since LGR5 is linked with epithelial-mesenchymal transition (EMT) in normal stem cell, we addressed whether EMT happened in the depletion of IKKα. Finally, we found that depletion of IKKα decreased the expression level of the mesenchymal markers (N-cadherin, Vimentin and β-catenin), while the epithelial markers (Claudin, ZO-1 and E-cadherin) decreased in the knockdown of IKKα (Figure 7D). Taken together, these findings indicate that the interplay between STAT3 and IKKα plays vital roles in BCC.

## DISCUSSION

The reports from different groups indicate that there is an opposed effect of IKKα in several types cancer in carcinogenesis [29–35]. Recently, we show that IKKα is diversely expressed in keratinizing and non-keratinizing carcinomas in the same type of cancer [36]. Here we firstly provide evidence that IKKα is absence in SCC of skin while is expressed in BCC, indicating the opposed roles of IKKα in skin cancer between SCCs and BCCs, which are origin from both keratinocyte tumors. Moreover, we provide evidence that IKKα promotes EMT that is an unintentional behavior of cells during cancer progression [37, 38]. One significant difference between SCC and BCC is that SCC development is generally associated with cell dedifferentiation that IKKα is reversely involved in the process.

The cellular origin of BCCs, the great majority of skin cancer, arises from hair follicle stem cells, mechanosensory niches and multiple epithelial progenitor populations [39–41]. In the skin, multiple stem cell populations in the hair follicles and epidermis maintain tissue homeostasis and contribute to organ regeneration; furthermore, cancer stem cells (CSCs) have been purified and characterized from mouse multiple hair follicle [40, 42]. Dysregulated hedgehog (HH) signaling is a hallmark of BCC, and cilia have a dual role in mediating both the activation and the repression of the HH signaling pathway [17, 43]. LGR5, a marker of intestinal stem cells, maintains hair follicle stem cells by autocrine HH signaling [2]. We show that LGR5 protein is expressed in both BCCs and SCCs, moreover LGR5 is up-regulated by high level of IKKα in BCCs. Similar findings have been reported increasing LGR5 mRNA levels in BCCs using real-time quantitative RT-PCR analysis [3, 44]. However, we did not found that side population cells increased significantly in A431 cells (Figure S6).

Beyond HH signaling pathway, recently other signaling pathways such as STAT3 signaling pathway involve in BCCs carcinogenesis [45, 46], during the development of ultraviolet (UV) B skin tumors, STAT3 plays a critical role in both survival and proliferation of keratinocytes [47]. In addition, JAK-STAT pathway is possibly involved in BCC pathogenesis from expression-associated single-nucleotide polymorphisms using genome-wide association study of BCCs [26]. Our findings further demonstrated that activated STAT3 involved in BCCs carcinogenesis through targeting LGR5 promoter directly, meanwhile, several inflammation factors also stimulated the activation of STAT3 signaling pathway. Meanwhile, IKKα located in the nucleus and bound to the promoters of inflammation factors, in turn, increasing the expression levels of these factors, which could act in a paracrine manner around their environments. The signaling pathways between NF-κB and STAT3 cooperate to promote the development and progression of colon, gastric and liver cancers as a central signaling hubs in inflammation-mediated tumors [48–50]. Here we provide evidence that both IKKα and STAT3 involved in LGR5 expression in BCCs, while inflammation factors linked with the interplay.

Recent reports show that receptor binding by LIF1, IL-6 and related cytokines engages both gp130-Yes-YAP module and gp130-Src-Yap axis, stimulates self-renewal of cultured embryonic stem cells and links with inflammation to epithelial regeneration respectively [51, 52], indicating the potential interplay of signaling pathway between gp130-STAT3 and YAP-hedgehog (HH) module. In BCC, we first provide evidence the interplay role of gp130-STAT3 pathway on LGR5, a direct target of HH signaling pathway, is stimulated by IL-6 and other members of the IL-6 family member and a direct link between HH-LGR5 and gp130-STAT3 exist in BCC.

In addition, IKKα comprises a nuclear localization signal and therefore also confers important nuclear functions. In the nucleus, IKKα forms an intact complex with CREB binding protein (CBP) and contributes to NFκB promoted gene expression through phosphorylation of histone H3 [27, 28, 53]. Additional tumor-promoting nuclear functions of IKKα include cell cycle regulation and chromosomal accessibility as a chromatin modifier [54, 55]. Here, we show that the intact complex of IKKα and STAT3 could regulate the LGR5 and inflammatory factors in BCC. Moreover, we show that IKKα might control EMT through LGR5 pathway.

In summary, based both on the expression level of IKKα in skin-related cancers and on the impact of LGR5 on BCC tumorigenesis, we conclude that IKKα is not expressed in skin SCC but in the nucleus of skin BCC and skin related non-malignant diseases. Activation of STAT3 signaling pathway contributes LGR5 expression in dependent of IKKα. Knockdown of IKKα in cancer cells inhibits tumor growth and transition of epithelial stage to mescheme stage. Our findings provide that the interplay between STAT3 and IKKα plays vital roles in BCC.

## MATERIALS AND METHODS

### Antibodies, plasmids, siRNAs, chemicals and cell cultures

Primary antibodies ZO-1, E-cadherin, Vimentin, β-catenin, Ki67, gp130, IKKα,STAT3, p-STAT3(Y705) for IHC analysis and Western Blot were purchased from Cell Signaling (Danvers, MA). Primary antibody for LGR5 and β-actin were purchased from Sigma-Aldrich (St. Louis, MO). Primary antibodies for LGR6 was purchased from Millipore (Temecula, CA).

The TRC-shLSH lentivrus plasmids, shIKKα, shIKKβ, shIKKγ and LGR5 and Tet-on shIKKα were purchased from from Genechem (www.genechem.com.cn). The IKKα inhibitor (IKKi-II) was purchased from Selleck. The STAT3 inhibitor V, Stattic, was purchased from Millipore.

HaCaT, A431 and HEK293 cell line were purchased from the American Type Culture Collection (ATCC; Manassas, VA), and were cultured in DMEM (GIBCO, Life Technologies, Basel, Switzerland) medium with fetal bovine serum (FBS) to a final concentration of 10%. All cell lines were maintained at 37°C with 5% CO_2_.

### FACS

Flow cytometry was used to quantify cells in each phase of the cell cycle. Cells (2 × 10^5^) were plated into 6-well plates and treated with 40 μg/mL of IKKα inhibitor. Cells were harvested at 0, 24, and 48 h, and washed twice with PBS. Pellets were resuspended in 0.5 mL PBS, fixed in 4.5 mL of 70% ethanol, and incubated overnight at 4°C. To detect the fluorescent intensity of certain proteins, cells were counterstained in the dark with 50 μg/mL of phosphatidyl inositol (PI) and 0.1% ribonuclease A (RNase A) in 400 μl of PBS at 25°C for 30 min. Stained cells were assayed and quantified using a FACSort Flow Cytometer (Becton Dickinson, U.S.A).

### Wound-healing assay and MTS assay

The cultured cells were grown in 6-well plates until confluent. A scrape was made through the confluent monolayer with a plastic pipette tip of 1 mm diameter. Afterwards, the dishes were washed twice and incubated at 37°C in fresh RPMI containing 10% fetal bovine serum in the presence or absence of 40 μg/mL of IKKα inhibitor. At the bottom side of each dish, two arbitrary places were marked where the width of the wound was measured with an inverted microscope. Cell motility was expressed as the mean +/− SEM of the difference between the measurements at 0, 24, 48 and 72 h after wound.

Cell viability was measured using a CellTiter-Glo Luminescent Cell Viability Assay Kit (MTS) purchased from Promega Corp (Madison, WI) and used according to the manufacturer's protocol. At 24 h post-transfection with 50 ng/mL of EGF or 50 ng/mL of IL-6 in the presence or absence of 1 μg/mL Dox induced, cells were seeded into 96-well plates (2×10^3^ cells/well), and cell proliferation was recorded every 24 h for 4 days. Measuring the absorbance at 450 nm using a microplate reader assessed the number of viable cells.

### Transient transfection and luciferase reporter assay

We cloned a regulatory region of the human LGR5 gene. A 1077-bp (−754~+324) nucleotide sequence was amplified by PCR using the following primers: 5′-GGGGTACCTCTGTCACTCTGGCATCGATT-3′ and 5′-CCCAAGCTTAAGGACAGGAGCACACCGAGC-3′. Forward primer contained *Hind*III restriction site and the reverse primer harbored a *Kpn*I restriction site and a few additional nucleotides before the restriction sites. The PCR product was digested with *Hind*III and *Kpn*I, subcloned into the reporter vector pGL4.16 (Promega) to generate a reporter construct.

The LGR5 promoter luciferase reporter construct was created by cloning the LGR5 promoter (1077 bp) into the pGL4.16 vector (Promega), driving the expression of firely luciferase. For transfection, 800 ng pGL4.16 or putative LGR5 promoter luciferase plasmid and combinations of IKKα inhibitor were used along with pRL-SV40 (from Promega) plasmid for normalization of transfection efficiencies. 24 h after transfection, cells were harvested at 36 h after transfection and lysates were analyzed for luciferase activity using the Dual Luciferase Reporter assay (Promega) according to the manufacturer's directions with a GloMax™ Microplate Luminometer (Promega). The data represent the mean ± SD of the three independent experiments performed in triplicate.

To observe EGF, Stattic and CXCL1 afftecting the activities of LGR5, 24 h after transfection, cells were treated with 50 ng/mL of EGF (Invitrogen), 50 ng/mL of Stattic (Cell Signalling Technology, U.S.A.), or 50 ng/mL of CXCL1 (PreTech) for 24 hr. Cells were harvested at 48 h after transfection and subjected to the luciferase assay. Empty firefly reporter vector served as the negative control.

### Immunohistochemistry (IHC) analysis

Skin and related diseases biopsies, validated by pathologist Dr. Desheng Xiao (Xiangya Hospital), were obtained from Pathology Department of Xiangya Hospital. The skin tissue array was purchased from Pantomics (Richmond, CA, USA). IHC analysis of paraffin sections from skin tissues or xenograft samples was described previously [56]. The sections were incubated with antibodies as indicated. The images were surveyed and captured using a CX41 microscope (OLYMPUS, Tokyo, Japan) with the Microscope Digital Camera System DP-72 (OLYMPUS, Tokyo, Japan) and differentially quantified by two pathologists who were from the Second Xiangya Hospital, Changsha, China.

IKKα staining was considered positively by ascertaining cytoplasmic and nuclear expression. The determination result was obtained from semi-quantitative classification according to 10 more visual fields (×200). The slides were first scored as 0 (negative), 1 (buff), 2 (pale brown), and 3 (tan). Positive expression of IKKα were scored as 0 (negative), 1+ (< 10% of positively-staining tumor cells), 2+ (11-50% of positively-staining tumor cells), 3+ (50-75% of positively-staining tumor cells), and 4+ (> 75% of positively-staining tumor cells. Both the scores by multiply were regarded as the determination result.

### Quantitative real-time PCR

Cells were harvested with Trizol(Invitrogen). cDNAs were synthesized with SuperScript II (Invitrogen) according to the manufacturer's protocol. Real-time PCR analysis was performed using the Applied Biosystems 7500 Real-Time PCR System, according to the manufacturer's instructions. The reactions were performed in triplicate for three independent experiments: the results were normalized to β-actin. The primer sequences used were used in the following, EGF, mRNA forward primer, 5′- GCACCCTTCTTAATTTTCTCCCA -3′; reverse primer, 5′- TCTCTCTTGCCTTGACCCATT -3′; Il-6 forward primer, 5′- ACTCACCTCTTCAGAACGAATTG-3′, reverse primer, 5′- CCATCTTTGGAAGGTTCAGGTTG-3′; Cxcl1 forward primer, 5′- CGTGGCCACTGAACTGCG-3′, reverse primer, 5′-TTCCGCCCATTCTTGAGTGT-3′, and β-actin, 5′-CATGTACGTTGCTATCCAGGC-3′; reverse primer, 5′-CTCCTTAATGTCACGCACGAT-3′. The mean± SD of three independent experiments was shown.

### Chromatin immunoprecipitation (ChIP) assay

ChIP assays were essentially performed as previously described [57, 58]. ChIP DNA was analyzed by qPCR with SYBR Green (Bio-Rad) in ABI-7500 (Applied Biosystems) using the primers specified in the following, LGR5 forward primer: 5′- AGGTCTGGTGTGTTGCTGAG-3′, reverse primer: 5′- GAGTGACGTGGGGAAGTACT-3′; EGF forward primer: 5′- TCCTTTCTCTGCACTCCTGG-3′, reverse primer: 5′- GTGCACATTCCAGGAGCTTT-3′; IL-6 reverse primer: 5′- CTGGAGATGTCTGAGGCTCA-3′, reverse primer: 5′-CACCCCTCCCTCACACAG-3′; CXCL1 forward primer: 5′- AGGCAATGTTGGAAAACGCT-3′, reverse primer: 5′- TCGCCTGCAGATTGTTTAGC-3′. The antibodies used are as followed: IKKα (Santa Cruz), p-STAT3 (Cell Signaling, Danvers, MA), normal mouse IgG (Millipore).

### Nude mice and study approval

A xenograft tumor formation was essentially performed as previously described [58]. Mice were injected with A431 (2 × 10^6^ cells/mice) and their corresponding stable clones with knockdown of LSH expression *via* mammary fat pad. Mice with A431 cells were imaged from dorsal and ventral views once per week. Visible lung metastatic nodules were examined macroscopically or detected in paraffin-embedded sections stained with H&E. Data were analyzed using Student's t test; a *P* value less than 0.05 was considered significant.

All procedures for animal study were approved by the Institutional Animal Care and Use Committee of the Central South University of Xiangya School of Medicine and conform to the legal mandates and federal guidelines for the care and maintenance of laboratory animals.

### Statistics

The experiments were repeated at least three times. Results are expressed as mean ± SD or SEM as indicated. A 2-tailed Student's t test was used for intergroup comparisons. A *p* value less than 0.05 was considered statistically significant (*p < 0.05, ** *p* < 0.01, *** *p* < 0.001).

## SUPPLEMENTARY MATERIALS FIGURES


