# Supplementary material for: LGR5 expression is controled by IKKα in basal cell carcinoma through activating STAT3 signaling pathway
Source: Oncotarget. 2016 Mar 30;7(19):27280–94. doi: 10.18632/oncotarget.8465 (PMC5053649; doi:10.18632/oncotarget.8465)
Supplement: Supplementary file 1 [file oncotarget-07-27280-s001.pdf]

LGR5 expression is controled by IKKα in basal cell carcinoma through activating STAT3 signaling pathway

Supplementary Material

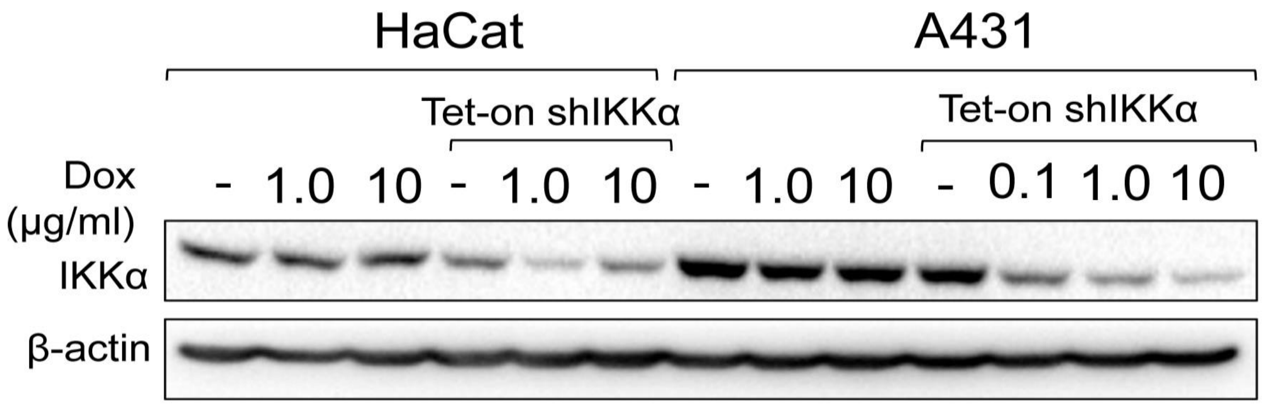

Figure S1. Western Blot analysis indicated the IKKα protein levels in the Tet-on sh IKKα cells.

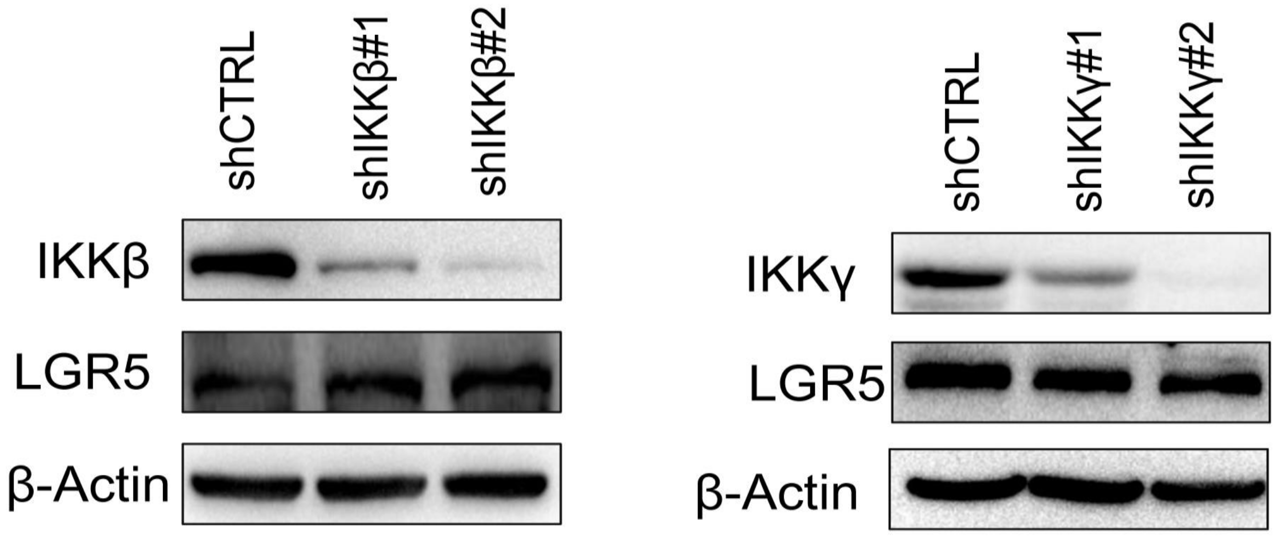

Figure S2. Western Blot analysis indicated the LGR5 protein levels after the knockdown of IKKβ (Left) and IKKγ (Right) in A431cells.

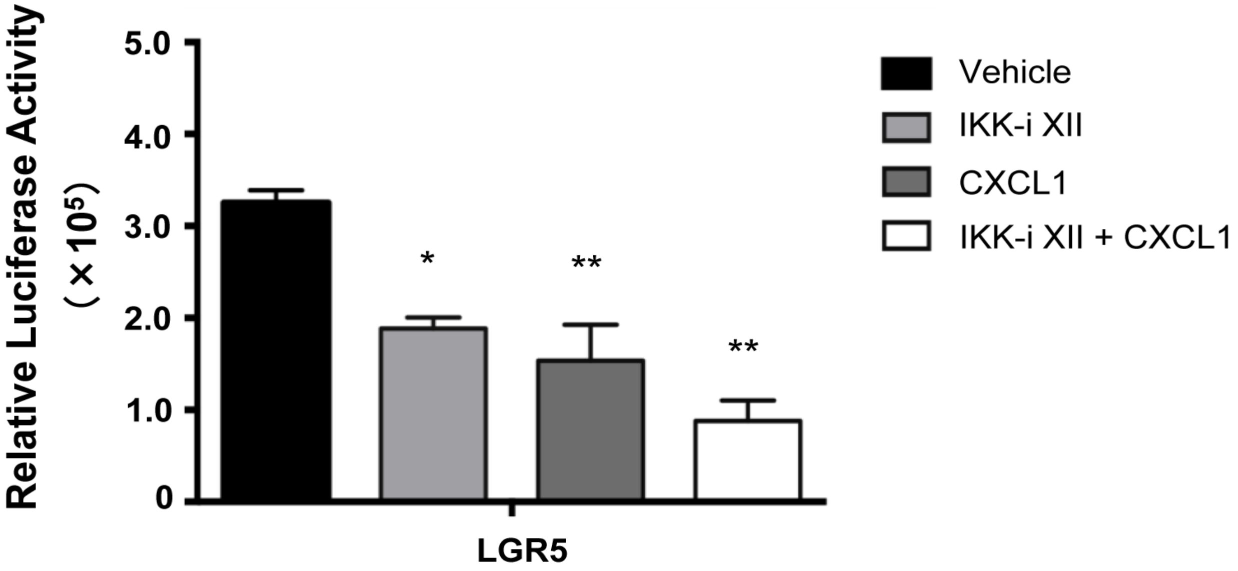

Figure S3. A luciferase reporter assay was carried out to evaluate LGR5 promoter activity in 293 cells with the treatment of IKKi-II and Cxcl1. All promoter luciferase intensity was normalized to the pRL renilla luciferase control reporter.

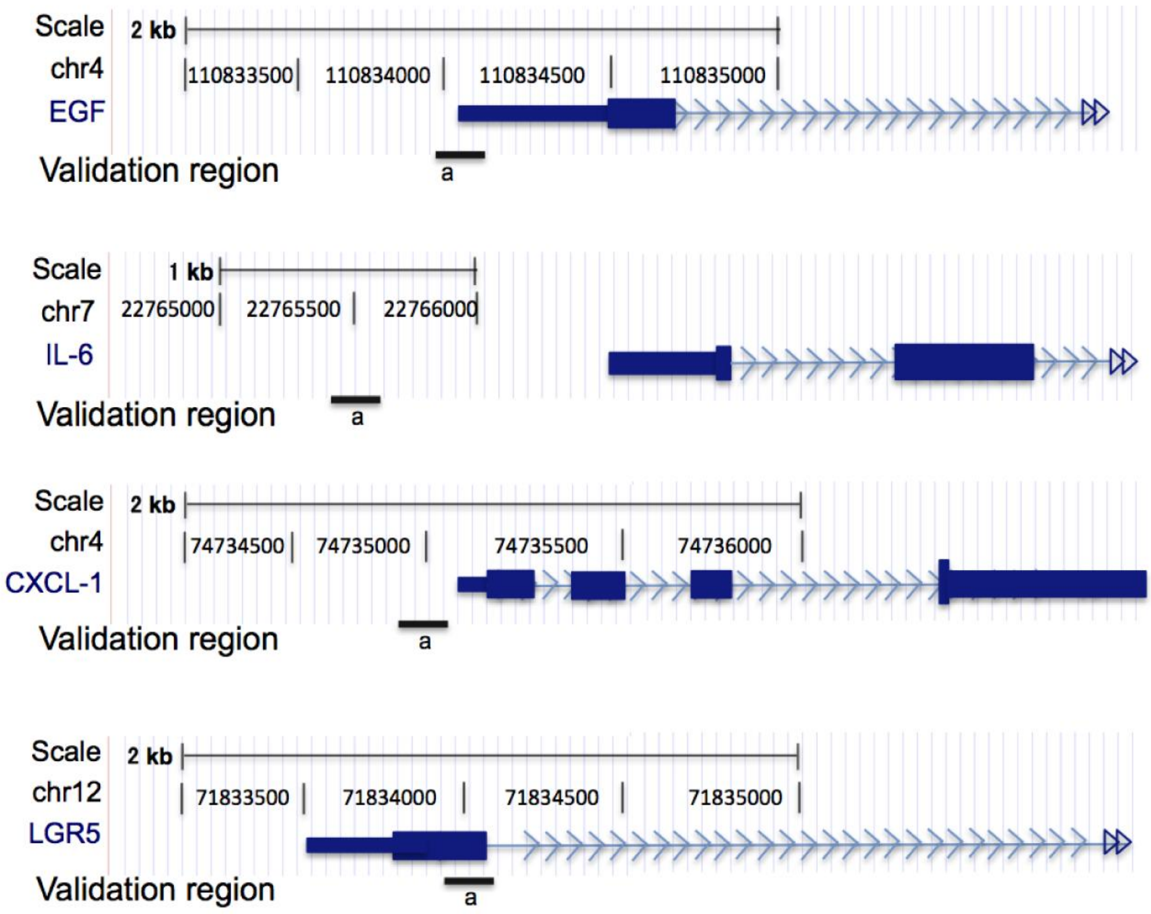

Figure S4. Schematic figures for primer sites of ChIP-PCR at indicated genes according to the UCSC genome browser (2009 GRCh37/hg19).

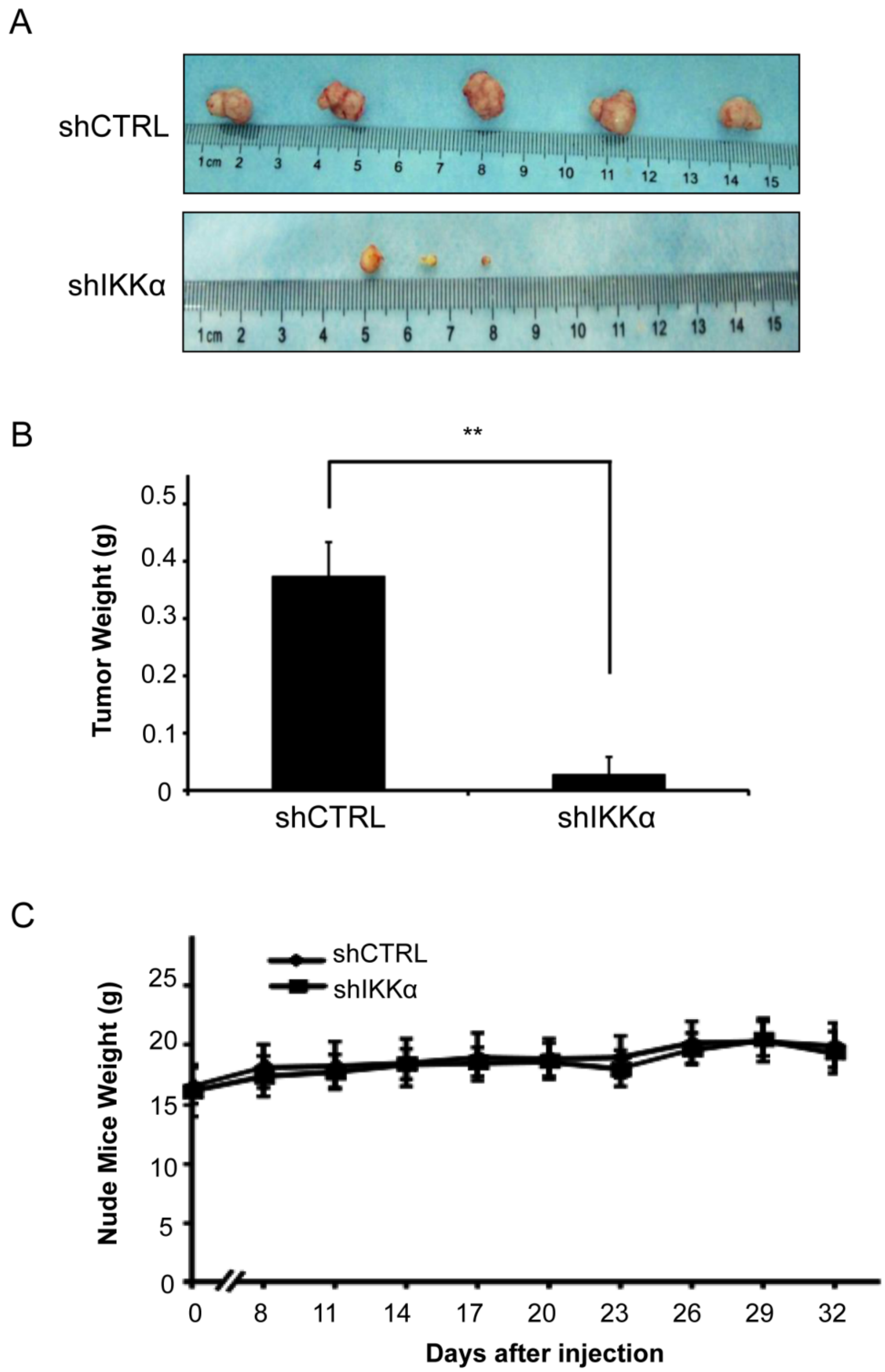

Figure S5. Inhibition of IKKα reduced tumor formation *in vivo*. A xenograft model of tumor growth was established in nude mice to evaluate the ability of A431 cells with a stable knockdown of IKKα to form tumors with 1×10^6 cells for 33 days. Tumor formation (A), tumor weight (B) and mice weight were recorded. ** p <0.01.

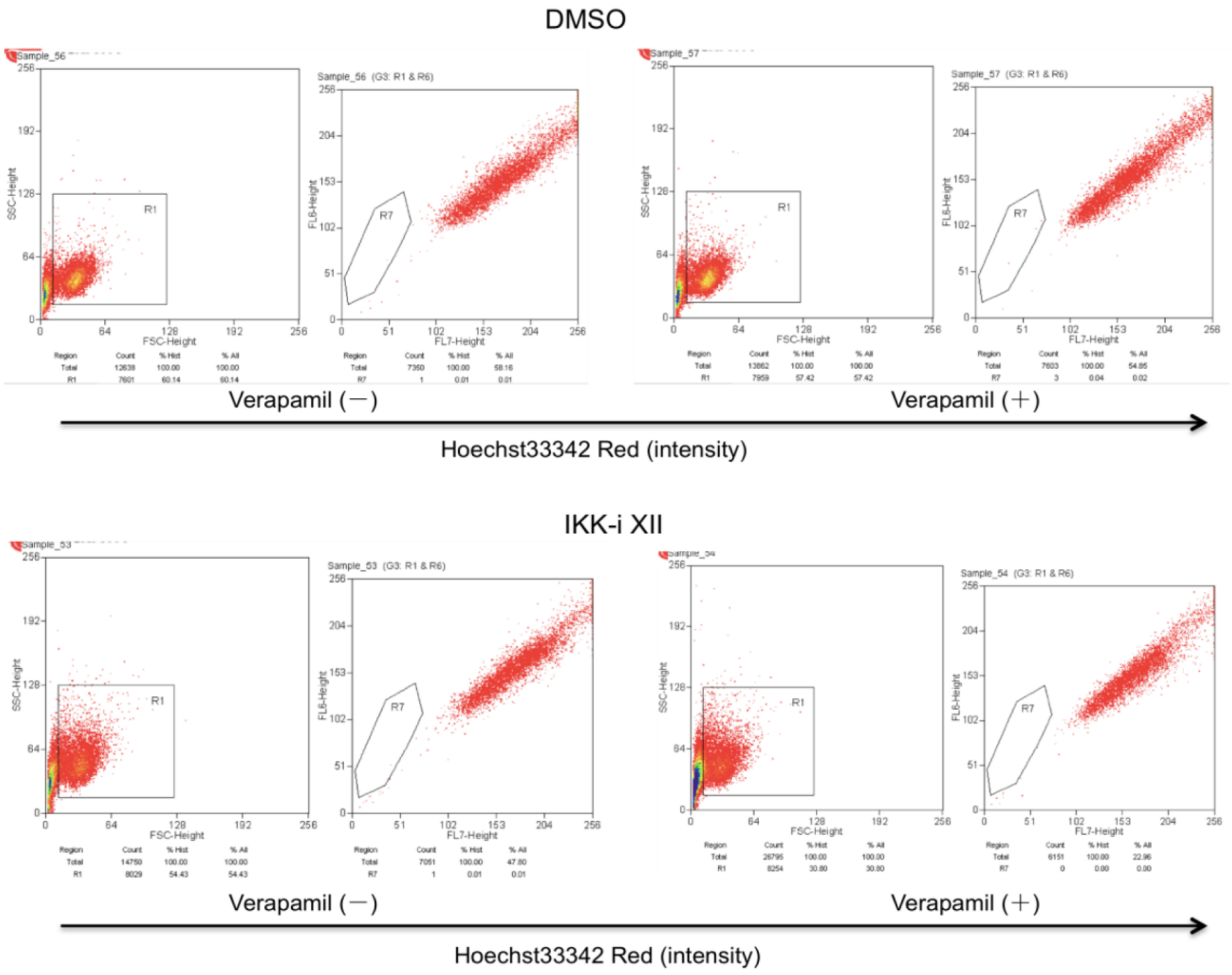

Figure S6. Side population analysis was used in A431 cells.
